# Chemical and Antimicrobial Effects of Air Non-Thermal Plasma Processing of Fresh Apple Juice with Focus on Safety Aspects

**DOI:** 10.3390/foods10092055

**Published:** 2021-08-31

**Authors:** Barbora Tarabová, Francesco Tampieri, Elisabetta Maran, Ester Marotta, Andrea Ostrihoňová, Marco Krewing, Zdenko Machala

**Affiliations:** 1 Faculty of Mathematics, Physics and Informatics, Comenius University, Mlynská Dolina, 84248 Bratislava, Slovakia; zilkovaandrea@gmail.com (A.O.); machala@fmph.uniba.sk (Z.M.); 2 Department of Chemical Sciences, University of Padova, Via Marzolo 1, 35131 Padova, Italy; francesco.tampieri@upc.edu (F.T.); elisabetta.maran92@gmail.com (E.M.); 3 Applied Microbiology, Faculty of Biology and Biotechnology, Ruhr University Bochum, Universitaetsstr. 150, 44780 Bochum, Germany; marco.krewing@rub.de

**Keywords:** apple juice, cold air plasma, food safety, pathogen inactivation, RONS, sugars, organic acids, polyphenols

## Abstract

Freshly squeezed apple juice was subjected to air non-thermal plasma treatment to investigate the capability of this processing method to inactivate microorganisms and to evaluate its safety when applied to liquid food products. Two different configurations of a transient spark discharge in ambient air were tested: an electrospray system with the juice flowing directly through the high voltage needle electrode, and a batch system, where the discharge was generated onto the surface of the juice. The key physico-chemical parameters of the juice, such as pH, conductivity, color, transmittance, and Brix degree, did not significantly change upon treatment. The concentration of nitrate ions formed by the plasma was safe, while that of nitrite ions and hydrogen peroxide was initially higher than the safety limits, but decreased within 24 h post treatment. The plasma effect on individual natural components of the juice, such as sugars, organic acids, and polyphenols, treated in water solutions led to their partial or substantial decomposition. However, when these compounds were plasma-treated altogether in the juice, they remained unaffected. The antimicrobial effect of the plasma processing was evaluated via the inoculation of model microorganisms. A stronger (6 log) decontamination was detected for bacteria *Escherichia coli* with respect to yeast *Saccharomyces cerevisiae*. Plasma processing led to a substantial extension of the juice shelf-life by up to 26 days if refrigerated, which represents a promising application potential in food technology.

## 1. Introduction

Conventional methods for food processing and the inactivation of food-borne pathogens are based on thermal treatments. This process is referred to as pasteurization when the temperature of the food product is temporarily increased up to 80 °C using Ohmic heating or dielectric heating methods. It is a traditional method that is used to ensure food safety as it is sufficient to inactivate various food-borne pathogens in different types of food products. However, particularly ultra-high temperature (UHT) processes may lead, besides the pathogen inactivation, to a reduction in the food quality (nutrition values, vitamin content, and changes in sensory properties, such as taste or color) due to the modification/degradation of heat-sensitive bioactive components [1]. The loss of nutrient properties is in contradiction with the fact that fresh fruit and vegetable products are sought for their health benefits. A growing customers’ trend for demanding long-lasting fresh products leads to the concept of “minimal processing” technologies. Therefore, new food-processing technologies that can achieve the required level of sterilization and safety without thermal input while preserving the nutrient and health benefits were investigated recently, e.g., pulsed electric field, high hydrostatic pressure, ultrasound, and ionizing radiation [2].

Non-thermal (cold) plasma known for its antimicrobial properties achieved without the requirement of excessive heat is predetermined for the applications in sterilization/pasteurization of fresh foods and food packaging. It was already successfully tested for pasteurization or processing of various food products, e.g., fresh fruits and vegetables [3,4,5], including fresh juices [6,7]; nuts [8,9]; meat and fish products [10,11,12]; and dairy products [13,14,15].

Cold plasma in contact with liquids can be generated using various electrical discharge set-ups. Dielectric barrier discharges (DBDs) [16,17,18,19,20,21], atmospheric plasma jets [22,23,24,25], glow discharges [26,27], pulsed discharges in liquids [28,29], or hybrid reactors with discharges above and in liquids [30] are the most explored discharge configurations for liquid food treatments, e.g., for juices. In recent years, a growing number of studies focused on microbial inactivation [16,21,24,30,31,32,33], shelf-life extension [16,26,34], pesticides or toxin degradation [20,35], or possible enhancement of the organoleptic properties and the composition [36,37,38,39] of different types of fruit or vegetable juices, including apple, orange, tangerine, blueberry, pomegranate, carrot, tomato, cherry, or coconut water, have appeared. The results are promising, however, the achieved level of microbial inactivation or the effects on the stability of juice components are variable and depend on the type of the discharge configuration, direct/indirect treatment type, and a wide range of treatment conditions (including the used gas, electrical parameters, and treatment time), as well as the type of juice and its composition (antioxidant activity, total soluble solid content, presence of metal ions, etc.) [6,7,24].

Considering the studies that focused on the cold plasma processing of apple juice, various species of food-borne pathogens and spoilage yeasts were successfully inactivated [18,19,21,24,25,28,30,31,40]. Generally, it was shown that the microbial inactivation increased with a longer treatment time and/or a higher applied voltage/power. The inactivation effect was attributed to the synergistic effect of the short- and long-lived reactive oxygen and nitrogen species (RONS) and the damage to the microbial cells at different levels due to the plasma-induced electrical or physical effects.

Besides the pathogen inactivation that helps to prolong the juice shelf-life time, an inactivation of undesirable endogenous enzymes is also beneficial for food preservation and extension of the food shelf life. Polyphenoloxidase (PPO) and peroxidase (POD) are the major enzymes that are responsible for the color change of apple juice due to enzymatic browning, which is a major problem that deteriorates the juice quality. Successful inactivation of these enzymes in apple juice was shown in works by Farias et al. [41] and Illera et al. [42]. 

As is already known, non-thermal plasmas generated in air or gases with admixtures of N_2_/O_2_ produce a large amount of various reactive oxygen and nitrogen species (RONS). These species, along with charged particles or UV radiation, play a dominant role in plasma inactivation effects [43]. RONS were considered the main antimicrobial agents that cause cell damage at different levels. They are also responsible for the enzyme inactivation and, thus, improvement in the shelf-life time. On the other hand, these plasma-generated agents and their chemistry may induce adverse effects on the juice composition and organoleptic properties (most RONS are strong oxidants). Furthermore, RONS produced in higher concentrations may be harmful to human health. Therefore, the impacts of cold plasma processing on food quality and biological safety must also be carefully considered. 

So far, a few studies have evaluated the effects of atmospheric cold plasma processing on the quality of fruit juices and addressed the concern regarding the safety aspects of this kind of plasma treatment [27,31,44]. Most of the studies focused on the plasma processing of apple juice investigated the effects of cold plasma on pH and titratable acidity, total soluble solids content (in °Brix degree), total phenolic content and antioxidant capacity, changes in color and browning, volatiles, identification of RONS, or chemical element analysis. Studies showed either no change in pH [19,24] or a decrease in pH that was linked with an increase of the titratable acidity [18,31,41] and typically minimal or no change in the total soluble solids content and reducing sugars [21,27,31]. A visual difference in the color (i.e., darkening/browning) of the apple juice was observed after plasma treatment [24,31,41,42], but no color change was observed by Dzimitrowicz et al. [27], where the apple juice was applied as a flowing liquid cathode. The observed changes were ascribed to the insufficient inactivation of the PPO and POD enzymes, but also to the oxidation reactions of the pigment compounds during the plasma treatment or storage period. 

Several authors observed an increase in the total phenolic content after plasma treatment that was typically linked with the increased antioxidant capacity [27,41,42]. This was attributed to the possible breakdown of cellular membranes of the dissolved plant material during the plasma treatment, where, according to Farias et al. [41], the phenolic content followed an inverse relationship with PPO enzyme activity. Lower PPO activities induced higher phenolic content because both PPO and POD use phenolic compounds as a substrate against oxidants. Wang et al. [21,40] studied the effect of plasma treatment on the volatile compounds of apple juice. The total concentrations of alcohols, esters, aldehydes, and ketones exerted no significant change after plasma treatment. Interestingly, Dzimitrowicz et al. [27] presented the only study up to now that focused on the possible cytotoxic properties of cold-plasma-treated apple juice on both the human normal intestinal epithelial cells and colon adenocarcinoma cells that may arise due to the possible change/degradation of the bioactive juice components. The results showed that the treatment using the direct current glow discharge with apple juice as a flowing liquid cathode did not inhibit the growth of both human cell lines after 24 h of observation. The plasma treatment had no harmful effect on the nutritional properties of the apple juice toward normal cells and preserved its bioactive components. Furthermore, only in the works of Liao et al. [31] and Xiang et al. [18] were the concerns about the nitrates and hydrogen peroxide concentrations regarding potential health risks addressed.

Therefore, for the future applications of cold plasma processing, it is important to design plasma processes that suppress the negative effects that potentially affect consumer preferences. Besides this, further investigations should include a more comprehensive analysis of plasma-initiated oxidation reactions with a focus on a deeper analysis of the stability of the quality-determining ingredients, mainly vitamins or polyphenols. Furthermore, the search for possible residues in plasma-treated liquid foods is required in order to verify their safety for human health.

The objective of the present work was to test the use of cold air plasma generated using a transient spark discharge in two different systems for the non-thermal processing of freshly squeezed apple juice. Apple juice was chosen because apples are one of the most consumed fruits for their health benefits. Furthermore, apple growing is widespread across Europe and, therefore, they are easily available. The main objective was to evaluate the pasteurization efficacy and safety of the plasma processing of the juice. We focused on the production of the reactive oxygen and nitrogen species, mainly nitrites, nitrates, and hydrogen peroxide, that may represent a possible health risk for consumers. Furthermore, we investigated the possible modification and degradation of the main organic juice components, i.e., organic acids, polyphenols, and sugars, due to the process of plasma treatment. We also focused on the effects of cold plasma on the nutrient and sensory properties of the treated juice, such as changes in pH, conductivity, color changes, changes in °Brix, and endogenous enzyme inactivation. While investigating all these juice properties, we furthermore examined the inactivation efficacy of the plasma treatment on the model bacteria *Escherichia coli* and yeast *Saccharomyces cerevisiae.*

## 2. Materials and Methods

### 2.1. Experimental Set-Up of Air Transient Spark Discharge for Juice Treatment

**Figure 1:**
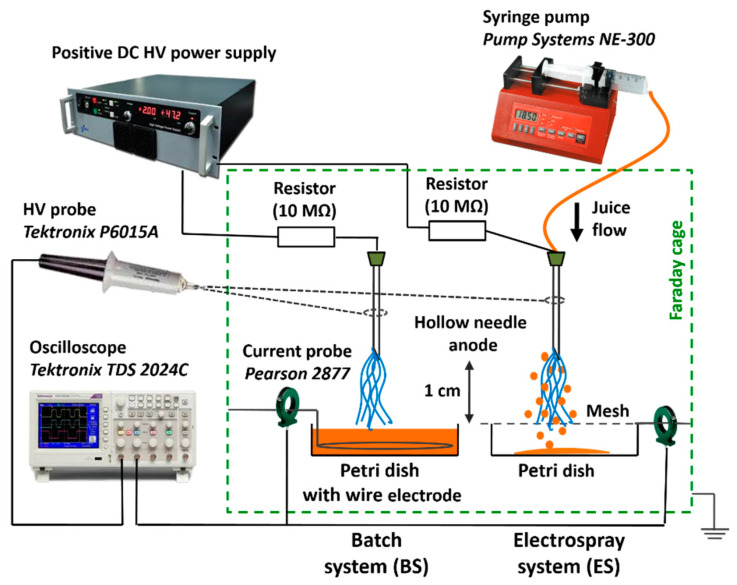
Experimental set-up of air transient spark discharge in batch (BS) and electrospray (ES) systems for juice treatment.

A DC-driven positive transient spark (TS) discharge in direct contact with the juice was generated in ambient air at atmospheric pressure in two different system set-ups, which are depicted in Figure 1. A transient spark discharge is a self-pulsing repetitive streamer-to-spark discharge with a very short duration of a strong spark current pulse with the repetitive frequency of ~1 kHz, which was described in detail in our previous work [45]. Both systems are based on a point-to-plane geometry using a sharp hollow needle as the high voltage anode made of stainless, chromium-nickel steel (Ø 0.8 mm × 40 mm, Sterican^®^, B.Braun, Melsungen, Germany). In the electrospray system (ES), the juice flowed directly through the needle with a constant flow rate thanks to the syringe pump (NE-300 Pump Systems Inc., Franklin, NH, USA) and it was collected into a sterile Petri dish (Ø 5 cm) below the grounded, stainless steel mesh electrode. Due to the high applied voltage, the effect of the electrospraying of the juice at the micrometric size (20–200 µm), droplets occurred. The ES set-up enabled the activation of the juice droplets that were sprayed directly in the discharge active zone in a similar way as previously investigated with water [46,47]. The batch system (BS) was based on the batch treatment using the TS discharge that was generated directly over the juice placed in the sterile Petri dish (Ø 5 cm) in which the grounded stainless steel wire electrode was submerged. The distance of 1 cm was kept in both systems between the needle tip and the mesh cathode (ES) or the juice surface (BS). Electrical discharge parameters were measured using a high voltage probe P6015A (Tektronix, Beaverton, OR, USA) and a Rogowski current monitor Pearson Electronics 2877 (USA). The typical applied high voltage was 11–13 kV for both systems with longer (~200 ns) current pulses of ~5 A amplitude for the BS and shorter (~50 ns) pulses of ~20–25 A for the ES. Despite these differences, the mean power for both systems was similar, in the range 1.5–2 W. Air TS generates cold non-equilibrium plasma at relatively low gas temperatures ~400–500 K [48,49], which does not increase the temperature of the treated liquids above room temperature and, therefore, thermal effects on the juice can be neglected.

### 2.2. Juice Preparation and Treatment Conditions

Ripped apples of the Ontario cultivar were harvested in the Bratislava region, Slovakia, and after being washed with tap water, they were juiced using a common juicer (MES3500, Bosch, Gerlingen, Germany). The fresh juice, still containing the pulp particles, was aliquoted into 50 mL sterile tubes and stored in the freezer at −20 °C. Before each type of experiment, the juice aliquot was thawed in a cold bath (approximately 15 °C), then centrifuged for 10 min at 3000× *g* (Hettich Zentrifugen, Tuttlingen, Germany) and filtered through a filter paper (grade 2) or 0.45 µM sterile syringe filters to remove all solid particles. Centrifugation and filtration are the minimal sample treatment procedures necessary before the injection of a complex sample into HPLC columns (see for example [50,51]) and are necessary to avoid scattering due to solid particles in spectrophotometric measurements. The filtered juice was treated using both systems under the following conditions: the juice flow rate was 1 mL/min in the ES, while 1 min treatment per 1 mL of juice was applied in the BS, with a typical volume of 5 mL of juice treated in each set-up. Both the treated juice and the control (untreated) were subjected to all chemical analyses and stored in the refrigerator at 4 °C to simulate the typical behavior of consumers, up to a maximum of four weeks. Control juice samples were processed in the same manner, except for the plasma treatment.

### 2.3. Detection of Reactive Species

Nitrite ions (NO_2_^−^), nitrate ions (NO_3_^−^), and hydrogen peroxide (H_2_O_2_) in the plasma-treated juice were measured using analytical methods that are typically used for plasma-treated aqueous solutions [46,48]. All reactive species were measured up to the seventh day post plasma treatment, while the juice was stored in the fridge at 4 °C. These methods were first tested for possible interferences due to the specific juice composition. To minimize the naturally high absorbance background in the visible region, the juice was diluted 20 times with deionized water before the chemical analyses. The dilution was taken into account when evaluating the concentrations or reactive species. Hydrogen peroxide was measured using the Amplex^®^ Red Hydrogen Peroxide/Peroxidase Assay Kit (Invitrogen, Waltham, MA, USA). In the presence of peroxidase, the Amplex Red reagent reacts with H_2_O_2_ in a 1:1 stoichiometry to produce the red oxidation product resorufin. This assay was performed spectrophotometrically. The absorption of resorufin was measured using a UV-VIS spectrophotometer (UV-1800 Shimadzu, Kyoto, Japan) at 571 nm with the extinction coefficient ε = 58,000 cm^−1^ M^−1^. Nitrite and nitrate ions were measured using Griess reagents [48]. This colorimetric method is based on the reaction of NO_2_^−^ with the Griess reagents under acidic conditions, which resulted in a deep purple azo compound with an absorption maximum at 540 nm. We used the Nitrate/Nitrite Colorimetric Assay Kit #780001 (Cayman Chemicals, Ann Arbor, MI, USA) and the assay was performed spectrophotometrically (UV-1800 Shimadzu, Japan). NO_3_^−^ ions were converted into NO_2_^−^ using the nitrate reductase enzyme and subsequently analyzed the same way as the NO_2_^−^. Concentrations of H_2_O_2_, NO_2_^−^, and NO_3_^−^ were calculated based on standard curves. 

### 2.4. pH, Conductivity, Transmittance, and °Brix Measurement

As the production of reactive species in plasma-treated liquids is linked with the changes in pH and conductivity, we measured the changes in pH (pH 3110, WTW, Oberbayern, Germany) and electrical conductivity (GHM 3430 Greisinger Electronic, Regenstauf, Germany) of the juice before and after the plasma treatment for up to one week. For the juice characterization, the Brix degree (°Brix) sugar content measurements based on the refractive index of non-treated and plasma-treated juice was performed before and after the plasma exposure. Transmittance in the VIS region (350–700 nm) of non-treated and plasma-treated apple juice was measured using a UV-VIS spectrophotometer (UV-1800 Shimadzu, Japan), also up to one week post plasma exposure. 

### 2.5. Quantification of the Organic Components of the Juice

Organic components of the juice were detected and quantified by means of three different high-performance liquid chromatography (HPLC) systems. Identification was carried out based on literature data [50,51,52,53,54,55,56] and by comparison with standard solutions. Sugars were analyzed using an HPLC 1260 Infinity (Agilent Technologies, Santa Clara, CA, USA) equipped with a refractive index detector (RID) and a Luna^®^ NH2 (Phenomenex, Torrance, CA, USA) column (250 × 4.6 mm i.d., particle size 5 μm). The eluent was an 80:20 CH_3_CN:H_2_O mixture and the elution was isocratic at a flow of 3 mL/min. The column was thermostated at 40 °C, apple juice samples were diluted 20 times before injection, and the injection volume was 20 μL. Organic acids were analyzed using an HPLC (Thermo Scientific, Waltham, MA, USA) instrument (P2000 pump with UV6000LP Diode Array Detector) with a Zorbax SB-Aq 150 × 4.6 mm i.d. column (Agilent Technologies USA) with a particle size of 3.5 μm and an injection loop of 20 μL. The mobile phase consisted of a 2 mM phosphate buffer (pH 2) containing 1% acetonitrile. The chromatographic run was isocratic with a flow rate of 1 mL/min and the elution was followed at 210 nm. Polyphenols analysis was carried out with an HPLC system (1100 series, Agilent Technologies, USA) connected to a diode array (G1312A) and a mass spectrometer detector (MSD SL Trap) equipped with an electrospray source (ESI) and an ion trap as the analyzer. A Kinetex^®^ C18 (Phenomenex, USA) as a column (150 × 4.6 mm i.d., particle size 5 µm) and H_2_O + 0.1% HCOOH (solvent A) and CH_3_CN + 0.1% HCOOH (solvent B) as eluents were used with the following gradient program for B: 5–45% in 20 min, to 100% in 10 min, 100% for 2 min. The flow rate was set at 1 mL/min and the injection volume was 20 μL. 

Chemical standards were used to identify and quantify, through external calibration curves, sugars ((d-(+)-glucose (99%, Carlo Erba Reagents, Milan, Italy), D-(+)-sucrose (98%, Sigma-Aldrich, Burlington, MA, USA), fructose (99%, Carlo Erba Reagents, Italy)), organic acids (L-ascorbic acid (99%, Sigma-Aldrich, USA), L-(-)-malic acid (99%, Janssen, Beerse, Belgium), citric acid (99.8%, Carlo Erba Reagents, Milan, Italy), D-tartaric acid (99%, Sigma-Aldrich, USA), D-(−)-quinic acid (98%, Sigma-Aldrich, USA)), polyphenols (phloridzin dihydrate (99%), chlorogenic acid (≥95%,), and (‒) epicatechin (≥98%,) from Sigma-Aldrich, USA) in the apple juice.

### 2.6. Peroxidase Assay

The enzymatic activity of the juice-borne peroxidase enzyme was evaluated using a pyrogallol-based assay in which pyrogallol was oxidized by a hydrogen peroxide-activated peroxidase, forming the brownish product purpurogallin [57]. The peroxidase assay was performed as follows. At first, the thawed juice was stirred for 1 h at 600 rpm with 1.5% (*w*/*v*) poly(vinylpolypirrolidon) (PVPP) (Sigma-Aldrich, USA) in an ice bath in order to scavenge the phenolic impurities in the juice. The insoluble fraction was removed using centrifugation at 4000× *g* for 20 min. PVPP-treated juice was kept on ice before the plasma treatment. The peroxidase assay was performed one hour post plasma treatment due to false-positive activity directly after plasma exposure. For the assay, all solutions were prepared fresh daily, kept on ice, and the pyrogallol solution was protected from light. First, 25 µL of 300 mM H_2_O_2_ (Central Chem, Bratislava, Slovakia) was mixed with 5 µL of 10% (*w*/*v*) pyrogallol (Central Chem, Slovakia) solution prepared in a 100 mM phosphate buffer (pH 6.5) directly in the microcuvette. Then, 70 µL of juice was added and the change of absorbance at 420 nm for 200 s was measured in the kinetic mode immediately. The linear slope at the beginning of the reaction was used to express the enzymatic activity of the peroxidase after the plasma exposure as a percentage of the residual enzymatic activity related to the enzymatic activity of the untreated juice, which was considered to be 100%.

### 2.7. Antimicrobial Effect of Transient Spark Treatment

We performed the experiments focused on the inactivation rate of model microorganisms: Gram-negative bacterium *E. coli* and yeast *S. cerevisiae.* For the juice inoculation, overnight cultures were prepared as follows: *Escherichia coli* ATCC 25922 (Czech Collection of Microorganisms, Brno, Czech Republic) was cultivated in Lauria–Bertani (LB) broth (10 g/L of casein peptone, 5 g/L of yeast extract, and 10 g/L NaCl (Biolab, Komárno-Nová Stráž, Slovakia)) with agitation (400 rpm) for 18 h at 37 °C; *Saccharomyces cerevisiae* S228C (obtained from the Department of Biochemistry, Faculty of Natural Sciences, Comenius University, Bratislava) was cultivated in YPD broth (10 g/L yeast extract (Biolife, Monza, Italy), 20 g/L peptone (Biolife, Italy), and 20 g/L d-glucose (Slavus, Bratislava, Slovakia)) with agitation (400 rpm) for 48 h at 30 °C. To keep the sterile conditions, the juice handling was performed in the hood adapted for the microbiological handling, i.e., sterile environment workplace with sterile equipment. Besides that, all small equipment necessary for the plasma treatment (i.e., syringes, tubes, Petri dishes, electrodes, etc.) were either new (sterile) or autoclaved (Tuttnauer 2340EK, Breda, The Netherlands) before use. Thawed juice was centrifuged for 15 min at 11,500× *g* (Hettich Zentrifugen, Germany) to remove the solid particles and potentially present native microorganisms. The purified juice was tested to confirm the absence of native microorganisms in the sample and then it was inoculated with either bacteria or yeasts to obtain a concentration of ~10^5^–10^7^ colony-forming units per mL (CFU/mL) and subjected to the plasma treatment. The plasma treatment was carried out under sterile conditions. The inactivation rate was measured for up to 26 days post plasma treatment. The juice samples (both control and plasma treated) were stored in the fridge in the sterile cultivation tubes for aerobic growth. The cultivation tests were carried out immediately post plasma treatment, and on the 2nd, 7th, 21st, and 26th days post plasma treatment. The number of viable microbial cells in the juice was evaluated by counting the CFUs cultivated on agar plates (LB agar or YPD agar) over 18 h at 37 °C (bacteria) or 48 h at 30 °C (yeasts) in triplicates. A set of independent experiments for each microorganism was repeated three times.

### 2.8. Data Analysis

All experiments were performed as a set of independent experiments with typically at least three repetitions and all measurements were performed in triplicates. The data were processed and evaluated using Origin 2018 (OriginLab, Northampton, MA, USA). The data are graphically plotted as a mean ± standard deviation (SD).

## 3. Results and Discussion

### 3.1. Production of Reactive Oxygen and Nitrogen Species in the Air-Plasma-Treated Apple Juice

**Figure 2:**
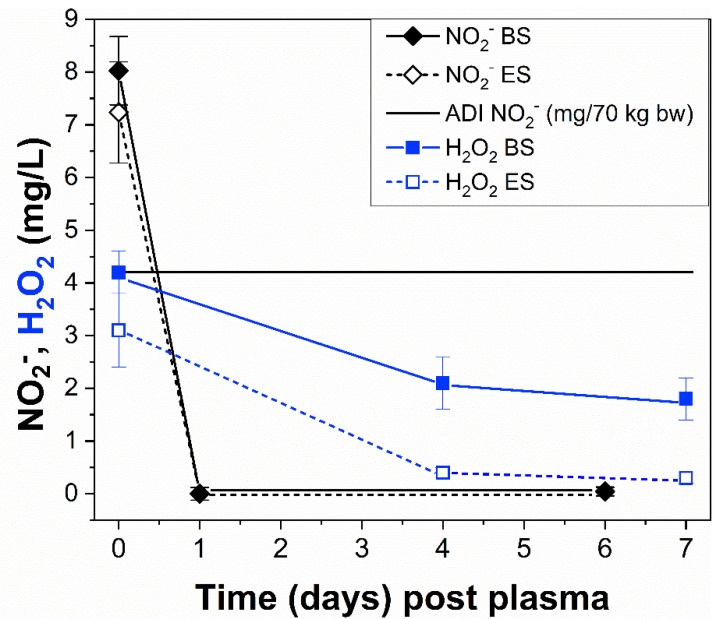
NO_2_^−^ and H_2_O_2_ concentrations (mean ± SD, *n* = 3) that was measured in the air-transient-spark-treated fresh apple juice compared to the ADI dose (NO_2_^−^), which was calculated for a human with a 70 kg body weight. The shown measured and ADI values are directly comparable assuming the daily consumption of 1 L of the juice per person.

Long-lived RONS (NO_2_^−^, NO_3_^−^, and H_2_O_2_) are typically produced in liquids that are treated using cold air plasmas. Concentrations of NO_2_^−^, NO_3_^−^, and H_2_O_2_ in the apple juice treated using both ES and BS under previously described conditions were monitored over one week post plasma treatment while the juice was stored at 4 °C. According to the regulations of the Scientific Committee for Food, the values of acceptable daily intake (ADI) per kilogram of body weight (bw) are defined as 0–3.7 mg of nitrate ions (NO_3_^−^) and 0–0.06 mg of nitrite ions (NO_2_^−^). These ADIs are applicable to all sources of dietary exposure [58]. The measured concentrations of NO_3_^−^ ions in the juice directly post plasma treatment (day 0) were already significantly lower (~10–11 mg/L) for both plasma systems than the maximal ADI (259 mg) calculated per average 70 kg human bw, assuming an exaggerated juice consumption of 1 L/day-person. In contrast, the concentrations of NO_2_^−^ ions immediately post plasma treatment were higher (~7–8 mg/L) than the calculated maximal ADI (4.2 mg)/70 kg bw, as shown in Figure 2. Nevertheless, the concentration of the measured NO_2_^−^ significantly dropped to zero 24 h post plasma exposure for both ES and BS and remained unchanged even after six days. It is worth mentioning that the ADI values were estimated with the margin of safety (MOS) of 500 for nitrate ions and 100 for nitrite ions.

Hydrogen peroxide is known as a bleaching or biocidal agent. Typically, it is present mainly in cosmetic products, e.g., hair products, or oral hygiene products (especially tooth-whitening products). H_2_O_2_ is not approved for use in foods in the EU (Regulation No. 1333/2008 on food additives), unlike in the United States, where it is generally recognized as safe to treat food under certain conditions (maximum treatment levels range from 0.04–1.25%) [59]. Therefore, no recommended ADI values are available for food products. However, there are regulations regarding its use in cosmetic or biocidal products, which can be applied to evaluate the safety of air transient spark plasma processing of the fresh apple juice. The measured concentrations of H_2_O_2_ in the juice directly post plasma treatment (Figure 2) were similar for both plasma systems, i.e., 4.2 mg/L for BS and 3.1 mg/L for ES. Further measurements on the fourth day showed a drop to a minimal concentration of 0.4 mg/L for the ES, which slowly decreased later on the seventh day (0.3 mg/L). The concentration for the BS dropped to only half of its initial concentration (2.1 mg/L) and did not significantly decrease, even on the seventh day (1.8 mg/L). The regulation on cosmetic products [60] defines the maximum concentration in ready-for-use preparations in oral products (such as toothpaste or mouth rinse) as 0.1% (i.e., 1 g/L) of H_2_O_2_ (present or released) and this concentration is considered safe. For the oral products, it is expected that a small amount of these products might be ingested on a daily basis. The no observed adverse effect level (NOAEL) was determined based on the repeated dose oral toxicity study on mice as 100 ppm, which represents an intake of ~20 mg/kg bw/day. The same NOAEL of 100 ppm of hydrogen peroxide in water corresponds to a concentration of 100 mg/L [61]. Considering these regulations, we can clearly see that the air transient spark treatment in both systems produced significantly lower concentrations of H_2_O_2_ in the apple juice while still considering an exaggerated juice consumption of 1 L/day-person. Furthermore, it is expected that H_2_O_2_ will be effectively already eliminated in the oral cavity by the endogenous cell protective mechanisms. 

### 3.2. Effect of the Air Cold Plasma on Selected Sensory Apple Juice Properties—pH, Conductivity, °Brix, and the Enzymatic Activity

The sensory properties of the juice (color, taste, smell) are indicated by the values of total acidity, pH, transmittance, enzymatic activity, or °Brix (sugar content). In general, any changes in the sensory properties are unwanted and, therefore, no or minimal changes should be induced by the food processing. The results regarding the effects of the plasma treatment on pH, conductivity, and °Brix are presented in Table 1.

First, we measured the pH and the conductivity (σ) of the apple juice, as these are the properties that are typically affected during the air cold plasma treatment of liquids. The pH and conductivity were measured immediately post plasma treatment and again after 6 days while the juice was stored in the fridge at 4 °C. We detected no change in the pH after the plasma treatment of the juice, even after 6 days post plasma exposure, whereas a slight increase of the conductivity (~0.1 mS/cm) was observed in the plasma-treated juice that later did not change after 6 days. Changes in pH and conductivity are in general linked with the production of aqueous reactive oxygen a nitrogen species [47,62]. The effect of plasma treatment on the pH is dependent on the type of the juice, the length of the treatment, and the plasma power that defines the production of the reactive species. Despite detecting nitrogen-containing acids (HNO_x_) in the plasma-treated juice that are typically linked with the change of pH, we expect that the complex organic composition of the juice acted partially as a buffer [17] and that the given treatment conditions (treatment time per milliliter of juice, power, etc.) were not sufficient enough to induce a pH change in the juice. No significant change in the pH of the cold-plasma-treated apple juice was also confirmed by other studies [19,24,40], while, for example, Xiang et al. [18] observed a significant drop in pH after 200 s plasma treatment and Illera et al. [42] observed a significant decrease in pH after a spark discharge treatment. This difference can certainly be attributed to the different plasma discharge set-ups, resulting in different production rates of acidifying reactive species.

The °Brix value or the total soluble solid content represented mainly by the sugars showed no significant change, i.e., the change was within the error bars. Similar results were published by Xiang et al. or Liao et al. [18,31] after the treatment of apple juice using dielectric barrier discharge cold plasma. For the °Brix measurement, we considered the partial evaporation effect during the plasma treatment. 

**Figure 3:**
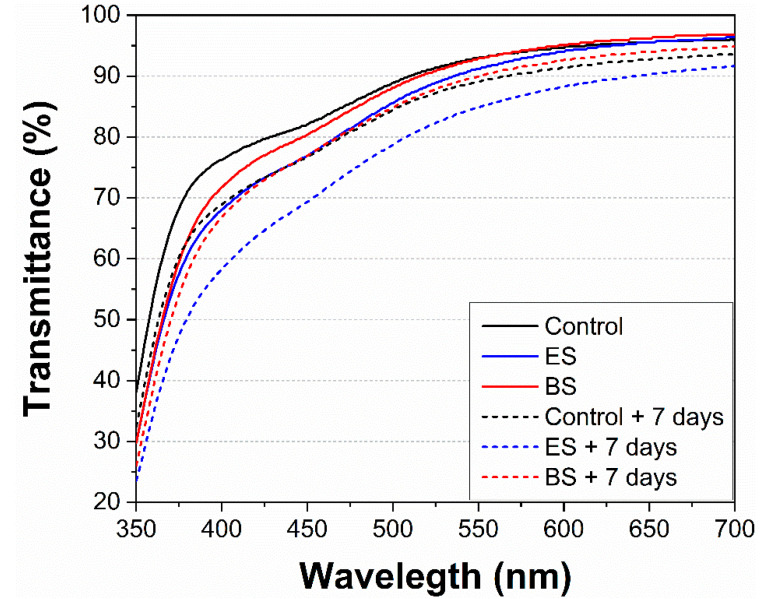
Comparison of the juice transmittance in the visible region immediately and 7 days post plasma exposure (+ 7 days) in the electrospray system (ES) and the batch system (BS).

**Figure 4:**
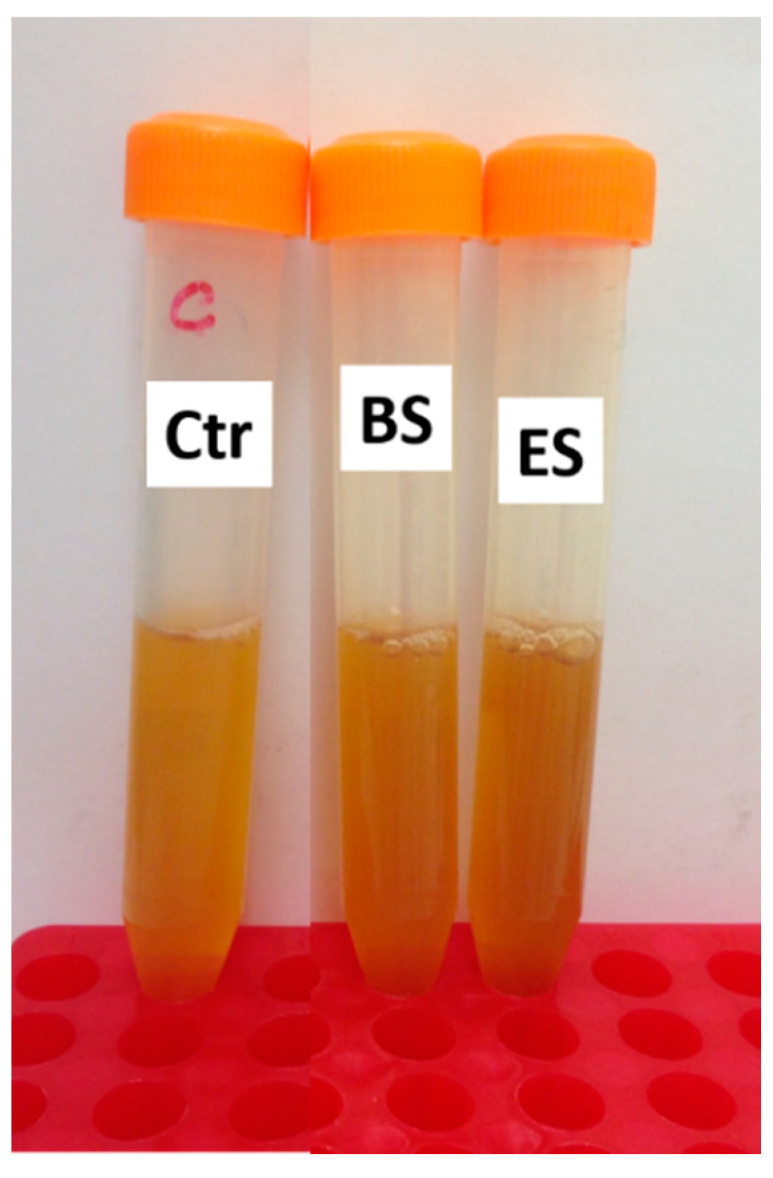
Typical samples of the non-treated (Ctr) and plasma-treated apple juice using a batch system (BS) and electrospray system (ES).

We measured the change in the transmittance and the activity of the peroxidase enzyme in the apple juice before and post plasma exposure as markers of the juice color. The transmittance was measured directly post plasma treatment in the visible region, i.e., 350–700 nm. Figure 3 shows a slight decrease (less than 10%) of the transmittance in the plasma-treated juice by both systems compared to the untreated juice, and a weaker effect was observed after the BS treatment. This change in the transmittance indicated a very mild browning of the juice, which was directly induced during the process of the plasma treatment, probably due to the oxidation of color pigments by the present reactive species [31]. For illustration, Figure 4 shows the photographs of the non-treated juice and the treated juices using both electrospray and batch plasma systems. A similar result (i.e., visual browning) was observed in most studies focusing on the color changes after cold plasma treatment of apple juice [24,31,41]. Furthermore, naturally occurring endogenous enzymes, i.e., peroxidase (POD) and polyphenol oxidase (PPO) are known to be responsible for the undesirable browning during the shelf-life storage of fresh fruit products, which lead to the loss of their quality [63]. We measured the enzymatic activity of the peroxidase enzyme in the non-treated and plasma-treated apple juice one hour post plasma treatment. As shown in Table 1, the plasma treatment using both systems resulted in a significant decrease in the enzymatic activity of the peroxidase enzyme, i.e., the remaining enzymatic activity represents ~47% for the ES and 29% for the BS (compared to the untreated juice which represents 100% activity). This decreased enzymatic activity should result in a slower browning process during the juice shelf storage. The stronger inactivation effect reached after BS treatment can be attributed to the higher production of RONS in this type of set-up, as shown in Figure 2. We repeated the measurement of the change in transmittance 7 days post plasma treatment (Figure 3). The decrease in the transmittance of the untreated juice up to ~8% occurred as a result of the natural browning due to the enzymatic activity. The decrease in the transmittance observed for the ES treated apple juice was similar (decrease up to ~9%). A weaker decrease in the transmittance (only up to ~4%) observed in the juice treated using the BS could be attributed to the stronger inactivation of the POD enzyme in comparison with the ES treatment. In addition, the effect of non-enzymatic browning was equally important, which was produced not only by the action of enzymes but also due to different reactions that occurred during food processing and storage [42]. Therefore, the natural browning process could be enhanced, despite the partial POD inactivation, due to this process of non-enzymatic browning induced by the plasma treatment alone and during the storage period. 

Interestingly, as shown by Illera et al. [42], the PPO enzyme inactivation process can continue and be enhanced in the period of storage of the juice post plasma treatment. However, we did not perform the POD activity measurement over the storage period and we cannot confirm that a similar effect would also be observed on the POD enzyme after treatment using a similar type of spark discharge. 

### 3.3. Effect of Cold Air Plasma on the Organic Components of the Apple Juice

**Figure 5:**
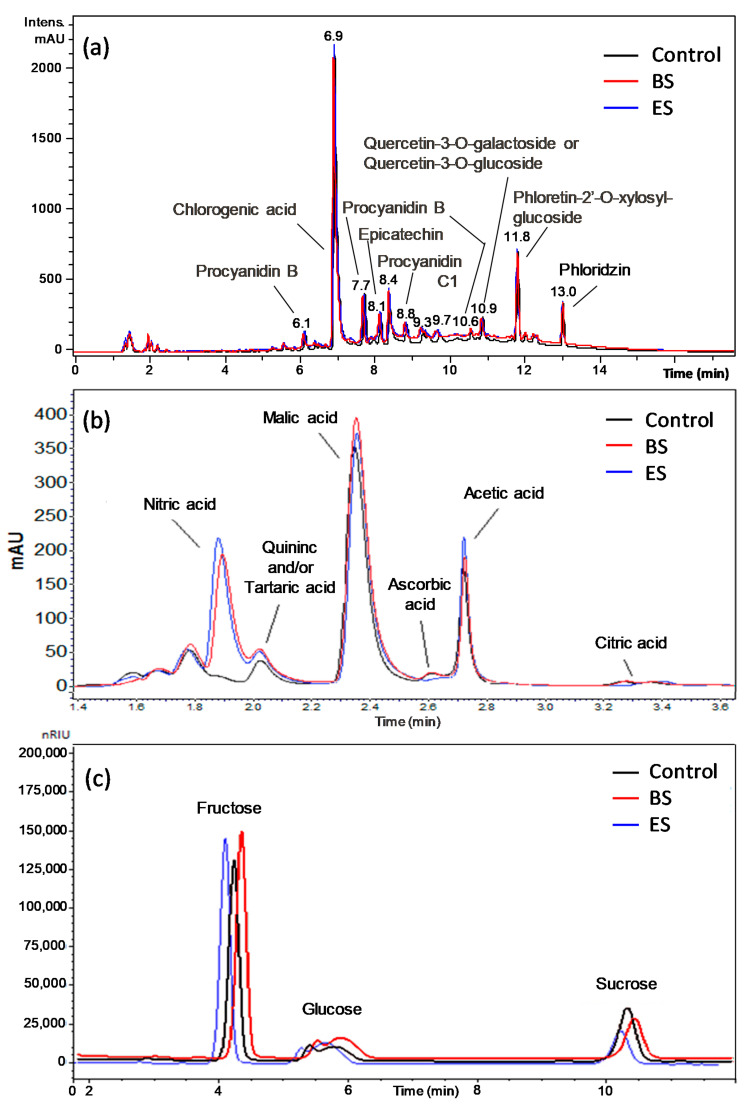
HPLC analyses of the untreated (control), batch system (BS)-treated, and electrospray system (ES)-treated apple juice samples using instrumental conditions to detect (**a**) polyphenols, (**b**) organic acids, and (**c**) sugars (see the Material and Methods, Section 2.5 for details).

Three different liquid chromatographic techniques were employed for the analysis of the sugars, organic acids, and polyphenols contained in the apple juice before and after the plasma treatments. Sugars and organic acids were identified by comparing the retention times of the peaks detected in the chromatographic runs with those of standard compounds by considering the species generally contained in the apple juice [50,54,55,56]. Polyphenol peaks were attributed based on their ESI-MS spectra and comparison with the literature data [51,52,53,54,55,56] (Figure 5a). Quantification was done for glucose, fructose, and sucrose, which were detected as the main sugars contained in the apple juice (Figure 5b). In the case of organic acids, the determination of ascorbic, citric, acetic, and malic acids was performed, while it was not feasible for tartaric and quinic acids due to the superimposition of their peaks (Figure 5c). In the case of polyphenols, three compounds chosen among the most abundant ones were quantified: chlorogenic acid, epicatechin, and phloridzin.

In Table 2, the average component concentrations measured in the apple juice and the percentage decrease detected after the BS and ES plasma treatments are reported. The results relative to the treated samples were adjusted to account for the evaporation occurring during the plasma processing. This was evaluated by weighting the juice before and after the treatment and was estimated as 7.9% in the batch treatment and 7.5% in the electrospray treatment. It was assumed that only water was lost from the samples. The concentration of sugars was substantially unchanged after the treatment. The slight decrease in the °Brix value (12.03 to 11.6) of the juice after the BS treatment could be attributed to the negligible decomposition of fructose (1%) and glucose (7%). Among the organic acids, the only significant change was due to the decomposition of ascorbic acid after the ES treatment, which was about 40%. Ascorbic acid, i.e., vitamin C, is the most easily oxidized vitamin in fruit juices. In general, it is a bioactive compound that is sensitive to different processing conditions, such as heat, the presence of oxygen, or UV [7]. A transient spark discharge generates high concentrations of O atoms, OH, and H_2_O_2_ [47] that could have been responsible for the decomposition of ascorbic acid thanks to the enhanced mass transfer in the ES. Chlorogenic acid was unaffected by the plasma treatment, while the concentrations of phloridzin and epicatechin, less concentrated than chlorogenic acid by about one order of magnitude, decreased, in particular, in the ES treatment.

Following the decomposition of some juice components, greater attention was paid to the possible formation of unknown degradation byproducts. For this reason, the plasma effect on the single components of the juice was investigated by treating aqueous solutions of these single components prepared in a phosphate buffer (21.6 mM) to obtain the same pH (3.2) and conductivity (1.97 mS/cm) as the apple juice. The concentration of each considered compound was chosen by taking into account its concentration measured in the apple juice. Thus, the following individual solutions were treated: glucose (0.2 M), fructose (0.2 M), sucrose (0.2 M), citric acid (10 mM), malic acid (10 mM), ascorbic acid (0.5 mM), chlorogenic acid (50 µM), epicatechin (50 µM), and phloridzin (50 µM). The observed decomposition rates after their individual plasma treatments are shown in Table 3. The concentrations of sugar solutions were unchanged, similarly to the case of the apple juice plasma treatment. When treated individually, citric and malic acids were preserved, both in the BS and in the ES treatments; however, ascorbic acid was completely decomposed in both cases. Chlorogenic acid, epicatechin, and phloridzin significantly decreased in concentration, while chlorogenic acid was the most sensitive to both types of plasma systems.

**Figure 6:**
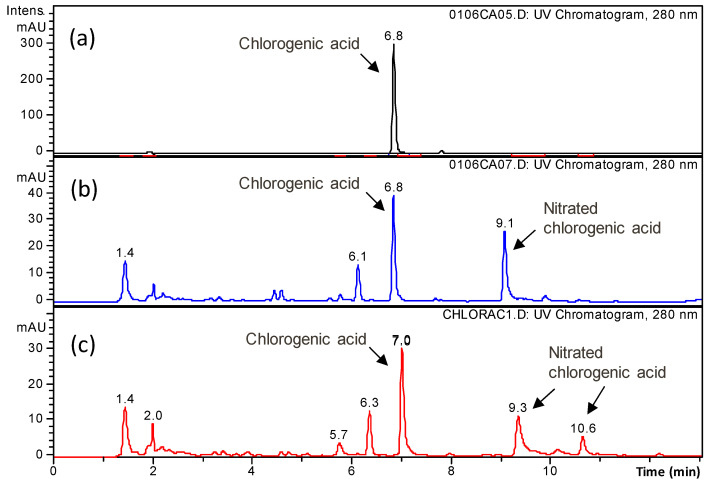
UV chromatograms (280 nm) of (**a**) untreated, (**b**) electrospray system (ES)-treated and (**c**) batch system (BS)-treated chlorogenic acid solution (5 × 10^−5^ M) in the phosphate buffer (21.6 mM, pH 3.2).

In the case of ascorbic acid, no products that were detectable under the same chromatographic analysis conditions employed appeared. In contrast, in the case of polyphenols, additional peaks due to the degradation products appeared in the chromatograms of the treated samples. In Figure 6, the UV chromatograms (280 nm) relative to the untreated and plasma-treated solutions of chlorogenic acid are shown as examples. From the MS spectra, two of the main peaks were ascribed to nitrated chlorogenic acid. In the case of treated phloridzin, hydroxylated and nitrated phloridzin were identified. In contrast, it was not possible to detect any degradation products of epicatechin.

**Figure 7:**
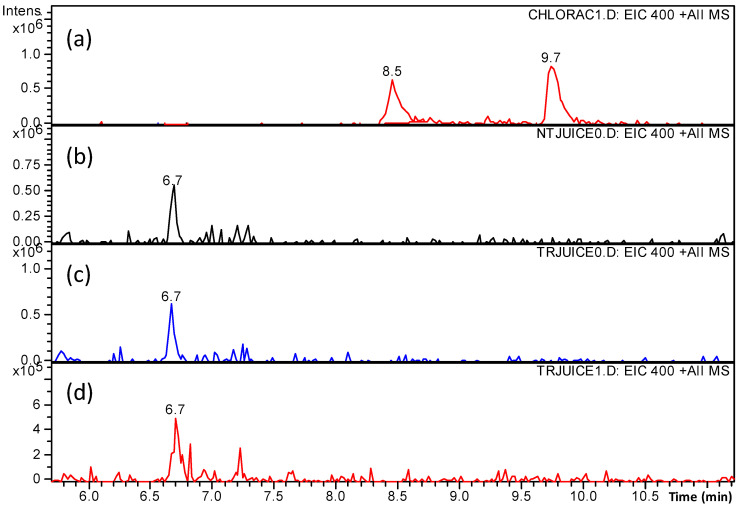
Extracted ion chromatograms of the signal at *m*/*z* 400 in the (**a**) batch system (BS)-treated chlorogenic acid solution, (**b**) untreated juice, (**c**) electrospray system (ES)-treated juice, and (**d**) BS-treated juice. The peaks at 8.45 and 9.7 min correspond to nitrated chlorogenic acid.

Before undertaking a more extensive investigation on the transformation products of ascorbic acid and polyphenols that were induced by the BS and ES plasma treatments, the hydroxylated and/or nitrated species of chlorogenic acid and phloridzin that were identified after the individual treatment of the two polyphenols were specifically examined in the samples of plasma-treated apple juice. Toward this aim, the signals of the ions originating from hydroxylated and nitrated polyphenols in the ESI-MS spectra were extracted from the total ion current acquired in the analyses of the treated juice samples. Assuming that the hydroxylated and nitrated species give the same instrumental response as their precursor, this procedure would allow for detecting a minimum concentration of 10^−7^ M. As shown in Figure 7, while two isomers of nitrated chlorogenic acid ([M + H]^+^, *m*/*z* 400, retention times 8.4 and 9.7 min) were detected when the chlorogenic acid solution was individually treated using plasma (Figure 7a), no signal attributable to nitrated chlorogenic acid was present in the ES-treated (Figure 7c) or BS-treated (Figure 7d) apple juice. Moreover, a compound with an identical *m*/*z* signal but different retention time (6.7 min) was observed both in the untreated (Figure 7b) and the treated juice (Figure 7c,d). Its presence both in the untreated and treated juice, combined with the fact that had a retention time that was different from nitrated chlorogenic acid, confirmed that it was due to a species with no relation to nitrated chlorogenic acid nor with the plasma treatment. Hydroxylated and nitrated phloridzin were similarly searched in the experimental data analysis and they also were not detected in the plasma-treated juice samples.

It can thus be concluded that the competition between many reaction targets in the juice medium reduced the effects of plasma RONS on the individual compounds, making them negligible.

### 3.4. Inactivation Rate of Model Microorganisms in Fresh Apple Juice by Air Cold Plasma

The European Commission Regulation on microbiological criteria for food [64] defines the safety of the manufacturing process of the unpasteurized fruit and vegetable juices (ready to eat) as satisfactory if the samples contain ≤10^2^ CFU/g of *E. coli* microorganisms or acceptable if two out of five batch samples contain 10^2^—10^3^ CFU/g and the rest of the samples contain ≤10^2^ CFU/g.

We tested the antimicrobial efficacy of the air transient spark plasma on the model microorganisms. The apple juice contaminated with a non-pathogenic strain of either *E. coli* or *S. cerevisiae* was treated in both electrospray and batch systems under previously mentioned conditions. The efficiency of cold plasma treatment on the inactivation rate (i.e., population growth) of model microorganisms was followed up to the 26th day post plasma exposure while the juice was stored in the fridge at 4 °C. The initial concentrations of microbial load were in the order of ~1 × 10^7^ CFUs/mL for *E. coli* and ~4 × 10^5^ CFUs/mL for *S. cerevisiae*.

**Figure 8:**
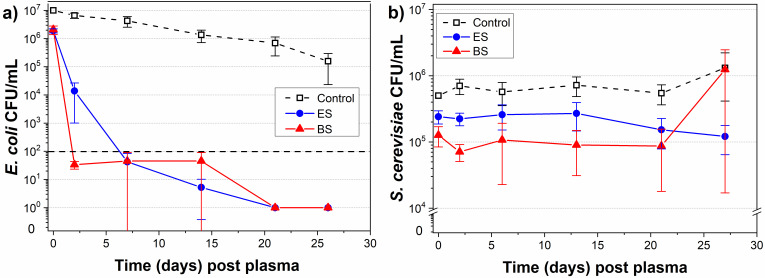
The post-plasma-treatment evolution in the electrospray system (ES) and batch system (BS) of the inactivation rate of (**a**) *E. coli* bacteria and (**b**) *S. cerevisiae* yeast with a high initial load in the apple juice, shown as a logarithmic scale (mean ± SD, *n* = 3). The dashed line in (**a**) represents the acceptable level of *E.coli* (≤10^2^ CFU/g) in unpasteurized fruit juices.

The results of the microorganism inactivation rates are presented in Figure 8. In both systems, the initial relatively low (<1 log) inactivation of *E. coli* immediately post plasma exposure was followed by a significant decrease in bacterial load within the first two days post plasma exposure (3 and 5 log reduction for ES and BS, respectively). This decrease reached a complete inactivation in both systems after twenty days and remained unchanged up to the 26th day (Figure 8a). The efficiency of inactivation of the yeast *S*. *cerevisiae* remained quite low over 26 days (~0.5 log), while slightly higher (~1 log) for the BS (Figure 8b). The transient spark in ambient air treatment with the electrospray system induced a very strong bactericidal effect due to the significant production of reactive species linked with the chemical changes in the treated solutions. Here we observed a much weaker immediate inactivation effect of *E. coli* bacteria (<1 log) than in our previous works with water (up to 7 logs), especially for the ES [46,47]. This difference was probably due to the shorter plasma exposure time per milliliter of juice and the specific juice composition, which seemed to inhibit the plasma inactivation effects in a similar way as the pH and chemical modification buffer. Despite this, the significant increase in the inactivation effect was observed 48 h post plasma exposure, which could be potentially attributed to the synergic effect of the acidic pH of the juice, the presence of the long-lived RONS, and the lower temperature during the juice storage, as was similarly shown by Surowsky et al. [25]. The relatively weak inactivation effect of the *S. cerevisiae* yeasts could be attributed to their different and more complex cell composition, which made them more resistant to the cold atmospheric plasma [30]. However, the high initial microbial load in our experiments (~10^5^–10^7^ CFU/mL) surpassed the naturally presented contamination by native pathogens by several orders of magnitude. As was shown by Shi et al. [16], the original load of native pathogens in the freshly squeezed orange juice was determined to be 3.20 × 10^2^ CFU/mL. A similar result was observed by Chutia et al. [33], who determined the aerobic microbial load in the tender coconut water to be 1.5–1.75 × 10^2^ CFU/mL. Hence, with these naturally lower microbial loads of 10^1^–10^2^ CFU/mL, we expected a much stronger plasma inactivation effect (especially for yeast inactivation).

The results of the inactivation of the microorganisms in apple juices using non-thermal plasma published up to date are summarized in Table 4, including the results obtained in this study. Based on the results achieved in each study on plasma-treated apple juices, it is obvious that the observed inactivation effects depend on the type of the discharge and the type and conditions of the juice treatment. The inactivation of both bacteria and yeasts in our study immediately post plasma treatment was much weaker than the results achieved by others (~1 log versus 4 to 7 logs). The main difference between ours and other studies was the type of apple juice. We used a ‘home-made,’ freshly squeezed juice, while the commercial or generic apple juice prepared from concentrate was typically used in other studies. In such types of juices, it is not obvious whether they underwent any kind of pre-treatment that could influence the inactivation results of microorganisms and result in stronger inactivation. Moreover, none of these studies observed the inactivation during a longer period post plasma treatment. We showed that despite the weak inactivation of bacteria immediately post plasma treatment, complete inactivation was achieved within the first week. 

Based on the results of other studies, it is clear that a direct type of treatment is necessary for stronger inactivation. Furthermore, the pulsed in-liquid system [28,30] or the surface DBDs with a spray reactor [19,40] seem to be the most effective in terms of strong inactivation of both bacteria or yeast, probably due to the effect of the electric field and the enhanced transfer of plasma-generated RONS into the juice. Undoubtedly, transient spark discharge used in our study employed both of these properties. It is a self-pulsed discharge producing short but strong pulses and is a rich source of gas reactive species that are transferred into the liquid [47], where this transfer is enhanced, especially in the ES. With respect to these facts, we attributed the enhanced inactivation effect in the post-plasma-treatment period to the synergy of the electric field that might cause the damage of the cell membranes and to the presence of RONS that caused the chemical damage of the cell membranes. Despite this, the achieved inactivation effect of the *S. cerevisiae* yeasts was much weaker in comparison with the studies of Wang et al. [19,21,40] or Vukušić Pavičić et al. [30]. However, when comparing the treatment time, Vukušić Pavičić et al. achieved the 7 log inactivation in only 9 min, while Wang et al. needed a 30 min long plasma treatment. Therefore, by employing the hurdle process, i.e., the combination of the mild pre-heating of the juice and the plasma treatment similarly to Vukušić Pavičić et al. is our plan to increase the efficiency of the yeast inactivation in future experiments.

## 4. Conclusions

In line with the current need for new non-thermal food pasteurization methods, we tested and compared non-thermal air plasma treatment of fresh apple juice using transient spark discharges in ambient air in two different electrical discharge setups. Besides demonstrating the antimicrobial effects of the plasma treatment tested on model bacteria and yeast and extending the juice shelf life, to the best of the authors’ knowledge, this is the first study that focused on the deeper examination of the organic chemical composition of the plasma-treated juice and potential safety aspects with respect to the production of RONS using the cold plasma treatment. The concentrations of residual typical plasma-generated RONS (NO_2_^−^, NO_3_^−^, and H_2_O_2_) in the treated juice were evaluated and were already well below the acceptably daily intake (ADI) values per kilogram of body weight 1 day post plasma treatment. In both setups, the investigated non-thermal plasma treatment of the apple juice did not significantly modify the overall chemical composition of the apple juice, namely, the sugars, organic acids, and polyphenols. In contrast, when ascorbic acid and polyphenols were treated individually in aqueous solutions with a pH, conductivity, and concentrations similar to those measured in the apple juice, they were partially or totally decomposed. This significant difference could be attributed to the competition among many components in the real juice for the same plasma-generated RONS, which limits their effect on each individual substance. Thus, while ascorbic acid, for example, was completely degraded if treated individually, it was less than halved when treated in the juice matrix. Moreover, possible by-products originating from the individual treatment of some polyphenols were successfully identified. Nevertheless, a subsequent chemical examination of the plasma-treated juice did not give any positive results, suggesting that potentially dangerous decomposition products were not formed (at a detectable concentration) during the plasma treatment of the juice. The approach based on the identification and searching of possible products of the plasma treatment was proposed as one of the strategies to verify the absence of any potentially dangerous by-products and to validate the non-thermal air plasma as a successful and safe technology for the shelf-life extension of fresh fruit juices. A focus on the assessment of the stability of the juice quality determining ingredients and a search for possible residues in the plasma-treated liquid foods is indeed an important requirement for all future investigations. This study represents a successful proof of principle at the laboratory scale. Upscaling the non-thermal plasma treatment into industrial technology is a general challenge to be addressed in the future.

## Figures and Tables

**Table 1:** Selected juice sensory properties of non-treated and air transient spark treated fresh apple juice in the electrospray system (ES) and the batch system (BS), which were measured directly post plasma exposure and after 6 days (mean ± SD, *n* = 3).

	Control	BS	ES	BS (+6 Days)	ES (+6 Days)
pH	3.23 ± 0.02	3.22 ± 0.01	3.23 ± 0.01	3.22 ± 0.01	3.21 ± 0.01
σ (mS/cm)	2.03 ± 0.01	2.12 ± 0.03	2.15 ± 0.03	2.10 ± 0.03	2.14 ± 0.03
Sugar content (°Brix)	12.03 ± 0.32	11.6 ± 0.37	12.03 ± 0.32	-	-
POD activity (%)	100	29 ± 6.1	46.9 ± 6.9	-	-

**Table 2:** Decomposition of the main organic juice components post plasma exposure (mean ± SD, *n* = 3) in the electrospray system (ES) and batch system (BS).

	Concentration	Decomposition (%)	
Sugars	Control (mM)	BS	ES
Fructose	369 ± 3	1	0.3
Glucose	90 ± 3	7	0
Saccharose	45.7 ± 1.3	0	0
Organic Acids	Control (mM)	BS	ES
Malic acid	80 ± 4	0	4
Acetic acid	57 ± 2	12	0
Citric acid	0.4 ± 0.03	0	5
Ascorbic acid	0.19 ± 0.04	10	42
Polyphenols	Control (µM)	BS	ES
Chlorogenic acid	475 ± 13	2	4
Epicatechin	36.7 ± 1.2	22	46
Phloridzin	29 ± 0.4	7	23

**Table 3:** Decomposition rate of single juice components (sugars, organic acids, polyphenols) after their individual plasma treatments (solutions with the given initial concentration were prepared in 21.6 mM phosphate buffer at pH 3.2) in the electrospray system (ES) and batch system (BS).

	Initial Concentration	Decomposition (%)	
Sugars	(mM)	BS	ES
Fructose	200	0.41	0
Glucose	200	4.3	3.9
Saccharose	200	3.0	0.2
Organic Acids	(mM)	BS	ES
Malic acid	10	0	0
Citric acid	10	0	0
Ascorbic acid	0.5	100	100
Polyphenols	(µM)	BS	ES
Chlorogenic acid	50	88	87
Epicatechin	50	38	48
Phloridzin	50	20	40

**Table 4:** Summary of the experimental studies that focused on the inactivation of microbes in apple juices using cold plasma, showing the types and conditions of the plasma treatment with the highest obtained antimicrobial effect.

Authors	Type of Apple Juice, Treated Volume	Plasma Source	Conditions of Treatment	Microorganism	Logarithmic Reduction	Ref.
Montenegro et al.	Generic supermarket brand, 0.8 mL	Pulsed in-liquid plasma system	Direct: 9000 V, f < 100 Hz, 4000 pulses	*Escherichia coli* O157:H7	7 log	[28]
Dasan et al.	Commercial clear juice, 11 mL	Atmospheric pressure plasma jet	Indirect: dry air (3000 L/h), 650 W, 120 s	*Escherichia coli* ATCC 25922	4 log	[24]
Liao et al.	Commercial juice, 3 mL	Dielectric barrier discharge plasma	Indirect: ambient air, 50 W, 30 s	mix of *E.coli* O157:H7, CICC 23429 and 10305	4.34 log	[31]
Surowsky et al.	Commercial juice (Granini), 2 mL	kINPen 09	Direct: Ar + 0.1% O_2_ (5 slm), 480 s	*Citrobacter freundii*	4.4 log	[25]
Xiang et al.	Commercial concentrated juice, 3 mL	Dielectric barrier discharge plasma	Direct: dry air, 90 W, 140 s	*Zygosaccharomyces rouxii* (GIM2.173)	5 log	[18]
Wang et al.	Commercially available juice, 500 mL	Gas-phase surface discharge plasma spray reactor	Indirect: dry air, 21.3 kV, 30 min	*Zygosaccharomyces rouxii* LB and IFO1130	6.8 log	[19]
Wang et al.	Commercial concentrated juice, 500 mL	Gas-phase surface discharge plasma spray reactor	Indirect: dry air (150 L/h), juice flow rate 9 L/h, 30 min	*Zygosaccharomyces rouxii* LB	5.6 log	[40]
Wang et al.	Commercial concentrated juice, 500 mL	Gas-phase surface discharge with active bubbling through liquid	Direct: dry air (150 L/h), 21 kV, 30 min	*Zygosaccharomyces rouxii* LB	5.6 log	[21]
Vukušić Pavičić et al	Commercial concentrated juice, 200 mL	Hybrid above and in-liquid (Ar 4 L/min) plasma	Direct: pre-heating to 40 °C, 120 Hz, 9 min	*Saccharomyces* *cerevisiae* ATCC 204508	6.6 log	[30]
Tarabová et al.	Fresh squeezed juice, 5 mL	Transient spark with electrospray or above the liquid surface	Direct: ambient air, 5 mL/1 min, 1 kHz	*E. coli* ATCC 25922, *S. cerevisiae* S228C	5–6 log 1 log	This publication

## Data Availability

The data presented in this study are available on request from the corresponding authors.
